# Circle-seq reveals that eccDNA may be a key blood biomarker for HBV-associated liver cancer

**DOI:** 10.3389/fgene.2024.1454153

**Published:** 2025-01-09

**Authors:** Xiao K. Ran, Xiao F. Zhao, Zhen W. Wei, Hua Z. Pang, Yan F. Tang, Rong Liu, Tie X. Wu, Xu D. Liu

**Affiliations:** ^1^ Graduate School, Guangxi University of Chinese Medicine, Nanning, China; ^2^ Hepatology Department, Ruikang Hospital Affiliated to Guangxi University of Chinese Medicine, Nanning, China; ^3^ Hepatology Department, Nanning Fourth People’s Hospital, Nanning, China

**Keywords:** biomarkers, expression profiles, target genes, whole transcriptome sequencing, extrachromosomal circular DNA, hepatocellular carcinoma, liver cirrhosis

## Abstract

**Introduction:**

Extrachromosomal circular DNA (eccDNA) regulates tumor occurrence and development. Relevant eccDNA profiles have been established for various types of cancer; however, the eccDNA expression profiles in the blood of patients with hepatocellular carcinoma (HCC) and liver cirrhosis (LC) remain unknown. The present study aimed to investigate the eccDNA expression profiles in the blood of patients with HCC and LC.

**Methods:**

Circle-seq was used to detect eccDNAs in the blood samples. Full transcript sequencing was used to analyze the RNA in the samples. Geno Ontology enrichment and Kyoto Encyclopedia of Genes and Genome pathway analyses were performed on differentially expressed eccDNA-related genes. The identified eccDNA is combined with mRNA to screen target genes using bioinformatics analysis. EccDNAs were confirmed through polymerase chain reaction and Sanger sequencing.

**Results:**

Overall, 103,235 eccDNAs were identified in HCC, whereas 67,110 eccDNAs were detected in LC. In total, 7,095 upregulated eccDNAs and 1,284 downregulated eccDNAs were identified. Following analysis of differential genes using bioinformatics, six candidate genes were screened out based on gene expression and cancer relevance. Experiments have verified that *LAMA4* ^*[circle112550019-112550510]*^ and *KANK1* ^*[circle674459-674907]*^ are real and expressed target genes, and their source genes are closely related to the survival time of patients with liver cancer.

**Conclusion:**

Our research results revealed the main characteristics of eccDNAs in the blood of patients with HBV-related HCC and LC. It was found that eccDNAs were mainly less than 1,000 bp in length. Difference analysis showed that some eccDNAs had consistent and overlapping expressions with mRNAs. We found that *LAMA4* ^*[circle112550019-112550510*]^ and *KANK1* ^*[circle674459-674907]*^ are target genes related to HCC, and both of them may become potential biomarkers for the diagnosis and prognosis of HCC.

## 1 Introduction

Oncogene amplification, a prevalent molecular alteration in cancer formation and progression offers a growth advantage to cancer cells by regulating key functional elements and overexpressing oncogenes, playing a pivotal role in tumor development (Beroukhim et al., 2010; Weischenfeldt et al., 2017). The formation of extrachromosomal circular DNA (eccDNA) is a common and potent mechanism for gene amplification, directly increasing the copy number of extrachromosomal oncogene DNA and accelerating tumor initiation and progression (deCarvalho et al., 2018). Moreover, eccDNA can enhance genetic heterogeneity within tumors through functional element enhancers (Turner et al., 2017), making it a highly specific tumor biomarker. eccDNA, a novel type of circular DNA located extrachromosomally, forms during DNA repair after damage, chromosome fragmentation, and specific DNA metabolic processes. It is characterized by its remarkable length, independent origin of replication, and autonomous amino acid encoding (Liao et al., 2020; Shoshani et al., 2021). Recent technologies, such as circular, whole genome, and chromatin open region sequencing, have been utilized to explore the eccDNA family in various cancer types, where it plays a vital role by amplifying genes and drug resistance (Turner et al., 2017; Kim et al., 2020; Kumar et al., 2020). The mechanism by which eccDNA functions as a mobile transcriptional enhancer to induce carcinogenic effects has been confirmed in various types of tumor diseases. This area of research is currently a popular and active topic of investigation (Sun et al., 2015; Cai et al., 2019; Lin et al., 2022). Moreover, eccDNA exhibits greater resistance to exonucleases, conferring greater stability compared to linear DNA (Zhu et al., 2017), rendering it an ideal biomarker that can be detected in blood circulation.

Hepatocellular carcinoma (HCC) is the most common form of liver cancer, characterized by poor prognosis and high mortality rate. Many cases of HCC are caused by persistent infection with hepatitis B virus (HBV) (Singal et al., 2020). HBV can easily evolve into cirrhosis if not treated in time, and it has a high probability of progressing to liver cancer, therefore it is important to find new diagnostic and therapeutic methods. Liver cirrhosis (LC) is an important pathological process that transforms various liver diseases into liver cancer. Many studies have tried to verify the association between liver cirrhosis and liver cancer (Keenan et al., 2019; Liu et al., 2022); however, their evolution mechanism has not yet been fully elucidated. There are few studies on eccDNA in liver diseases, and the expression and related mechanisms of eccDNA in liver cancer and cirrhosis are still unclear. Therefore, from the perspective of searching for specific biomarkers, we used a combination of multi-omics research and bioinformatics analysis technology to analyze the phenotypic characteristics of eccDNA and related differential genes in the blood of HBV-related HCC and LC. The aim of this study is to explore the mechanism of eccDNA on the occurrence and development of HCC, find new disease treatment targets, and provide theoretical basis for the development of new diagnosis and treatment directions.

## 2 Materials and methods

### 2.1 Blood sample

Ten patients were selected from Ruikang Hospital, affiliated with the Guangxi University of Traditional Chinese Medicine, from October to November 2022. HBV-DNA tests were all positive. This group consisted of 5 patients with HCC with an average age of 52.00 ± 4.00 years, diagnosed based on the “Guidelines for Diagnosis and Treatment of Hepatocellular Carcinoma (2019 Edition)” (Zhou et al., 2020). These patients were identified as HCC-1, HCC-2, HCC-3, HCC-4, and HCC-5. Additionally, 5 patients with LC, with an average age of 55.60 ± 12.38 years, were diagnosed in accordance with the “Guidelines for the Management of Liver Cirrhosis in China” (Xu et al., 2020). They were identified as LC-1, LC-2, LC-3, LC-4, and LC-5.

### 2.2 eccDNA sequencing analysis

The Circle-seq service was provided by CloudSeq Biotech Inc. (Shanghai, China) to profile eccDNAs in the blood of patients with HCC. Genomic DNA was extracted using the QIAamp DNA Blood Mini Kit (51,104; QIAGEN, Hilden, Germany). Then, circular DNA was separated from genomic DNA through the Plasmid Mini AX column (010-50; A&A Biotechnology, Gdynia, Poland). Column-purified DNA was incubated with FastDigest MssI (FD1344; Thermo Fisher Scientific, Waltham, MA, United States) at 37 °C for 16 h to digest mitochondrial DNA. The remaining linear DNA was removed by plasmid-safe ATP-dependent DNase (E3101K; Lucigen Corporation, Middleton, WI, United States) at 37°C in a heating block. Enzyme reactions were conducted continuously for 1 week, adding additional ATP and DNase every 24 h (30 units per day) according to the manufacturer’s protocol. Then, eccDNA-enriched samples were used as templates for phi29-based amplification with a REPLI g Midi Kit (150043; QIAGEN) at 30°C for 2 days (46–48 h), followed by purification using MinElute Reaction Cleanup Kit (28204; QIAGEN). Purified DNA was subjected to library preparation with GenSeq^®^ Rapid DNA Lib Prep Kit (GS-LC-004; GenSeq Inc., Shanghai, China). Sequencing was carried out on NovaSeq 6000 Sequencer (Illumina, San Diego, CA, United States) with 150 bp paired-end mode.

### 2.3 Whole transcript sequencing analysis

RNA high-throughput sequencing was used to detect RNA in the blood of patients with HCC and LC. RNA high-throughput sequencing was performed by CloudSeq Biotech Inc. (Shanghai, China). The experimental process was as follows: the GenSeq^®^ rRNA Removal Kit (GS-LC-010; GenSeq Inc.) was used to remove ribosomal RNA (rRNA) from the sample in accordance with the manufacturer’s instructions. The GenSeq^®^ Low Input RNA Library Prep Kit (GS-LC-032; GenSeq Inc.) was then used to construct a sequencing library according to the provided instructions. Subsequently, the quality and quantity of the constructed sequencing library were assessed using the Bioanalyzer 2100 system (Agilent Technologies, Palo Alto, CA, United States). Ultimately, 150 bp paired-end sequencing was performed using the Illumina NovaSeq6000 instrument (Illumina).

### 2.4 Data analysis

The original count of soft-clipped reads at the breakpoint was standardized using edgeR (v0.6.9) software. This process involved calculating multiples and *P*-values between the two sample groups to identify differentially expressed eccDNA (FC > 2 and *P* < 0.05). Differentially expressed eccDNA was subjected to clustering analysis using normalized counts via the heatmap 2 function in the R package. EccDNA visualization was achieved using IGV software (v2.4.10).

Bedtools (v2.27.1) was used to annotate genes for differential eccDNA and mRNA. The annotated differential genes were subjected to Gene Ontology (GO) functional and Kyoto Encyclopedia of Genes and Genomes (KEGG) pathway analyses. Differentially expressed eccDNA-related mRNAs were analyzed for GO function, gene annotation, and functional speculation. GO and KEGG entries with *P* < 0.05 were considered statistically significant.

### 2.5 eccDNA validation by polymerase chain reaction (PCR) and sanger sequencing

We conducted combined Circle-seq and full transcript sequencing to analyze differential genes. By integrating the biological functions and enrichment pathways of these differential genes, we identified six significantly expressed eccDNAs, significantly associated with liver cancer, and verified their expression in clinical samples. To confirm their authenticity, we randomly selected one sample from five HCC blood samples. DNA was extracted from this sample, and Accurate Taq Master Mix (Dye Plus) from Accurate Biotechnology (Changsha, China) was used for PCR amplification to generate PCR products and calculate their expression levels (Supplementary Table S1). The primers for eccDNA were designed using an “outward” directing strategy, and the PCR products were loaded onto a 1.5% agarose gel and visualized under an ultraviolet luminescence image analyzer (GE Healthcare Life Sciences, Chicago, IL, United States). Furthermore, we conducted Sanger sequencing using the PCR products, with gene sequencing services provided by CloudSeq Biotech Inc. The nucleotide composition of PCR products was compared between high-throughput and Sanger sequencing to verify the sequence of the cut site.

### 2.6 eccDNA source gene survival curve analysis

We obtained clinical data and gene expression levels of source genes from the Cancer Genome Atlas (TCGA) (https://www.cancer.gov) and the Gene Expression Omnibus (GEO) (https://www.ncbi.nlm.nih.gov/geo/) online databases. Subsequently, we conducted a gene survival analysis on eccDNA-derived genes.

### 2.7 Statistical methods

All statistical graphs and analyses in this study were performed using Prism v9.0 (Graphpad Software Inc., La Jolla, CA, United States) and IBM SPSS Statistics 24 (IBM Corp., Armonk, NY, United States). All values are expressed as mean ± standard deviation (SD). The significance of differences was determined using a one-way analysis of variance (ANOVA) or paired Student’s *t*-test. Statistically significant differences are indicated as follows: *P < 0.05, **P < 0.01, ***P < 0.001, ****P < 0.0001.

## 3 Results

### 3.1 Detection of eccDNAs in HCC and LC samples using traverse circle-seq analysis

**FIGURE 1:**
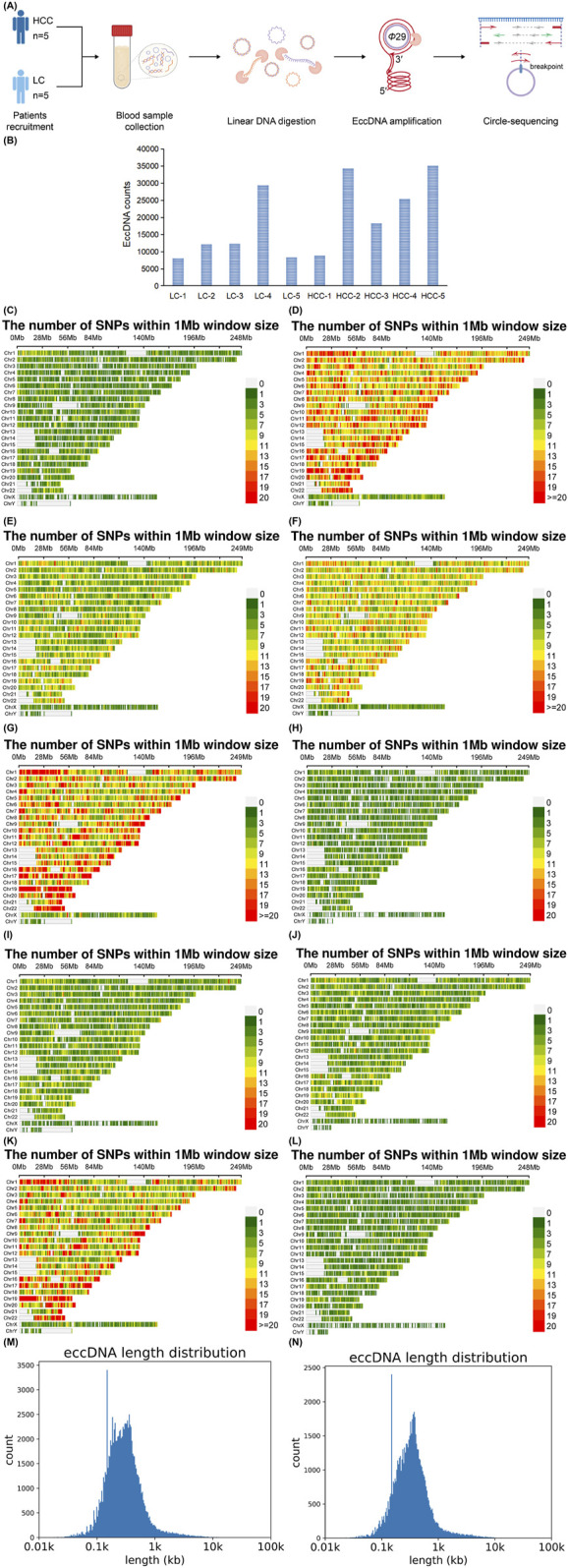
Sequencing method, total number, and length distribution of eccDNA. Circle-seq detects eccDNAs in the blood of individuals with HCC and LC. DNA is extracted from HCC and LC samples. Exonuclease is used to remove linear DNA, and the circular structure of eccDNA is opened through transposase treatment. Adapters are subsequently added to both ends, followed by repair with Klenow enzyme. The end of the product is nicked, and the product is amplified and purified for high-throughput sequencing research **(A)**. Total number of eccDNAs tested in each sample **(B)**. Variant genes enrichment per 1 MB on the 24 chromosomes in HCC and LC blood samples, HCC-1, HCC-2, HCC-3, HCC-4, HCC-5 **(C–G)**; LC-1, LC-2, LC-3, LC-4, LC-5 **(H–L)**. Distribution of all eccDNA lengths detected in HCC **(M)** and LC samples **(N)**. eccDNA, extrachromosomal DNA; HCC, hepatocellular carcinoma; LC, liver cirrhosis.

Circle-seq was used to analyze eccDNA in the blood of patients with HCC and LC (Figure 1A). A total of 103,235 and 67,110 eccDNAs were identified in HCC and LC samples, respectively. The eccDNA expression frequency in each sample was 8,874–35,108 in HCC and 8,061–29,399 in LC (Figure 1B), indicating abundant eccDNA expression in the blood of these patients. The number of enriched variant genes in HCC samples was generally greater than that in LC samples, which may be related to the formation of eccDNA (Figures 1C–L). The length distribution of eccDNA in both groups was 8–9,958,611 and 8–9,887,225 bp in HCC and LC samples, respectively (Figures 1M, N).

### 3.2 Characteristics of eccDNAs in HCC and LC samples

**FIGURE 2:**
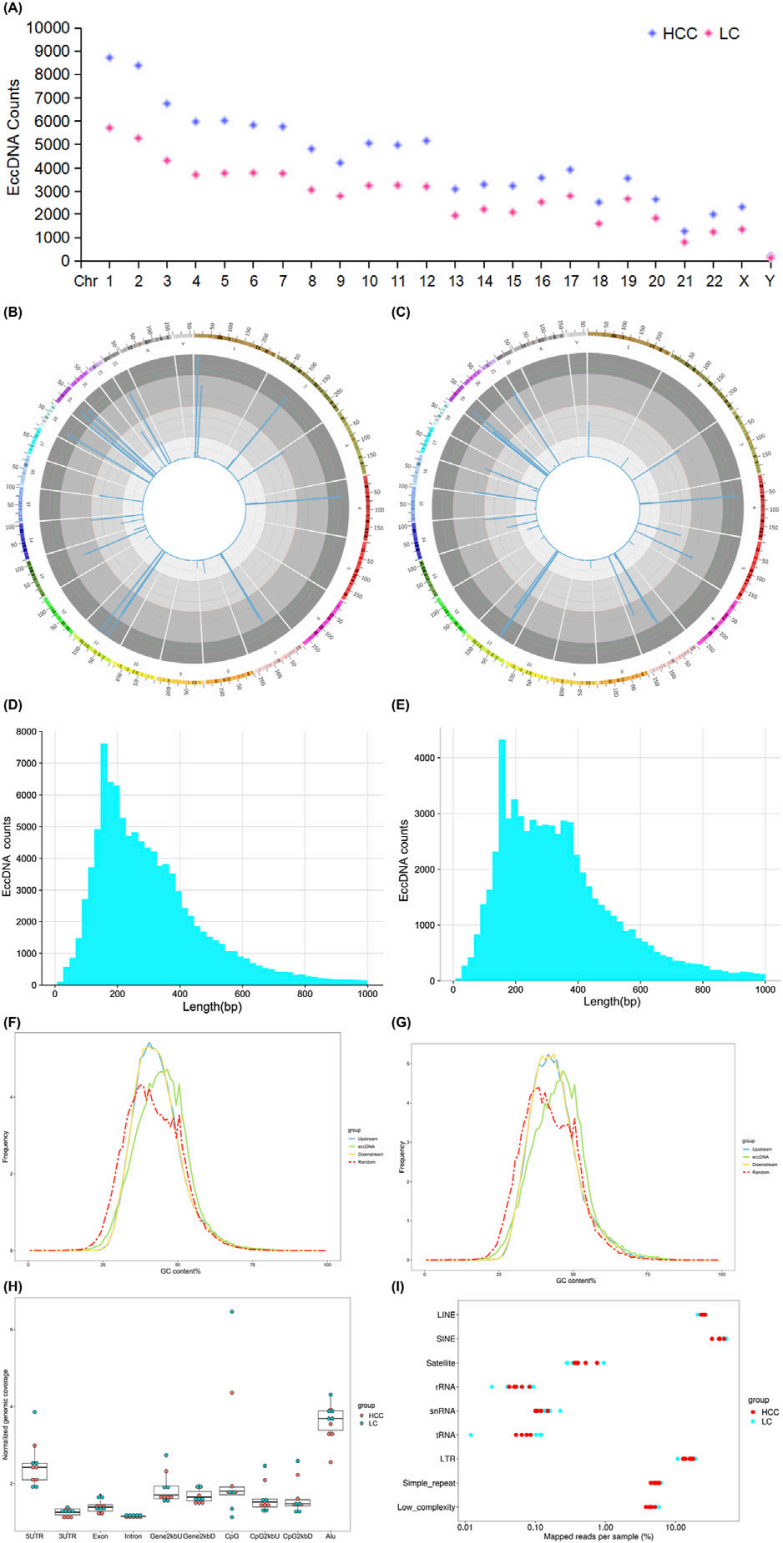
eccDNA characteristics detected in HCC and LC samples. Chromosomal distribution of eccDNAs **(A)**. Length distribution of eccDNAs in different chromosomes **(B, C)**. Length distribution of eccDNAs <1,000 bp in HCC **(D)** and LC samples **(E)**. GC content of the upstream and downstream regions of eccDNA in HCC **(F)** and LC samples **(G)**. Genomic element distribution **(H)** of eccDNAs from each sample, with red representing HCC and green representing LC. Total mapped repeat regions **(I)** of eccDNAs from each sample are represented in red for HCC and blue for LC. EccDNA, extrachromosomal DNA; GC, guanine-cytosine; HCC, hepatocellular carcinoma; LC, liver cirrhosis.

We examined eccDNA characteristics, including chromosome distribution, length distribution, guanine-cytosine (GC) content, and genomic distribution, in the blood of individuals with HCC and LC. First, we observed that these eccDNAs stem from all chromosomes (Figure 2A). In these two groups, we found no consistent correlation between gene-rich chromosomes and eccDNA formation frequency. For example, fewer eccDNAs originated from the gene-rich chromosome 19, whereas more eccDNAs were observed on chromosomes 1 and 2. Second, the size distribution analysis revealed that most eccDNAs were concentrated on chromosome 11 in both HCC and LC samples (Figures 2B, C). In HCC samples, eccDNAs with a length of <1,000 bp were the predominant types, accounting for approximately 95.50% of the total with a concentration in the 100–300 bp range. HCC samples comprised 98,588 eccDNAs <1,000 bp (Figure 2D). In LC samples, eccDNAs with a length of <1,000 bp were also the main subtype, totaling 63,195 eccDNAs (Figure 2E), accounting for approximately 94.17% of the total and ranging from 100 to 300 bp in length. Third, compared to other genomic regions, the GC content was more enriched in eccDNA sequences in HCC and LC samples (Figures 2F, G), indicating that elevated GC content is a common characteristic of eccDNA in HBV-related HCC and LC. Fourth, we explored the probable origin of eccDNA by mapping eccDNAs to various genomic (Figure 2H) and repetitive elements (Figure 2I). EccDNAs were particularly abundant in the 5′untranslated region (UTR) and the Alu region, and repetitive elements, such as long interspersed nuclear elements (LINE), short interspersed nuclear elements (SINE), and long terminal repeats (LTR), indicating that these regions produce eccDNA in HCC and LC more preferentially than gene-rich regions.

### 3.3 eccDNAs are differentially expressed in HCC and LC

**FIGURE 3:**
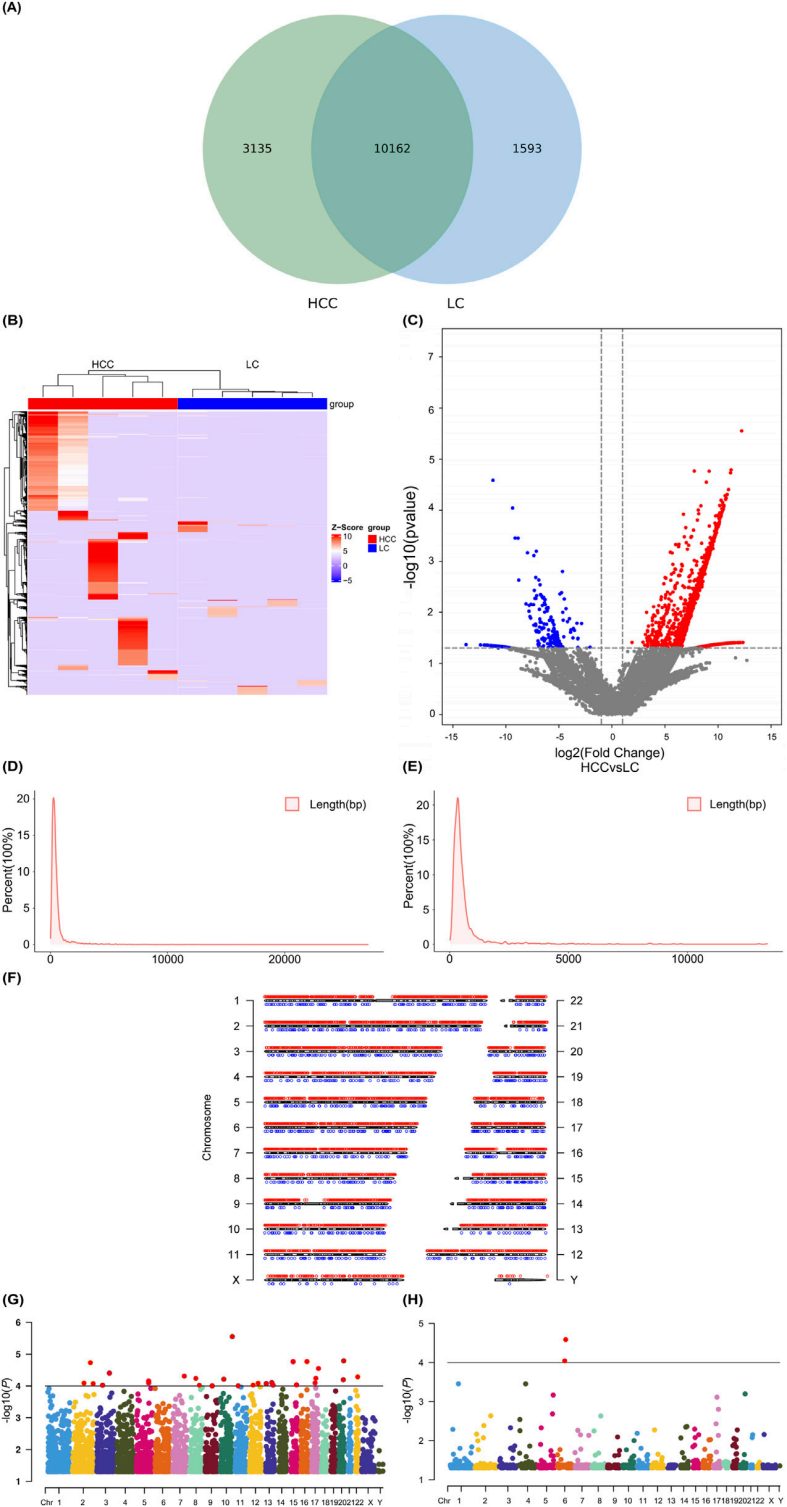
Expression analysis and distribution of differential eccDNAs related to HCC and LC. Venn diagram showing common and differential eccDNAs between HCC and LC **(A)**. Cluster heatmap showing differentially expressed eccDNAs in HCC and LC samples **(B)**. Volcano plot illustrating the differential eccDNAs, with blue dots representing downregulated genes, red dots representing upregulated genes, and black dots representing genes with no significant difference **(C)**. Length distribution proportion of upregulated differential genes **(D)**. Length distribution proportion of downregulated differential genes **(E)**. Chromosomal distribution of differential genes in HCC and LC samples, with blue dots indicating downregulated genes and red dots indicating upregulated genes **(F)**. Manhattan plot showing the correlation between the upregulated **(G)** and downregulated differential genes **(H)** related to eccDNAs on each chromosome and HCC. The higher the −log10 (*P*-value) of the gene locus, the stronger its correlation with HCC. eccDNA, extrachromosomal DNA; HCC, hepatocellular carcinoma; LC, liver cirrhosis.

A Venn diagram was used to express the commonality or specificity analysis of eccDNAs between the two groups of HCC and LC, showing that the two groups had 10,162 identical eccDNAs-related genes. The analysis of the differentially expressed eccDNA-related genes between the two groups showed that 3,135 and 1,593 eccDNAs were unique to the HCC and LC group, respectively. A total of 7,095 upregulated and 1,284 downregulated differential genes were screened out (FC > 2 and *P* < 0.05; Figures 3A, B). The volcanic plot visually represents the differential genes as upregulated and downregulated (Figure 3C). The length distribution of the upregulated differential genes ranged from 8 to 27,098 bp (Figure 3D), whereas that of the downregulated differential genes ranged from 20 to 13,346 bp (Figure 3E). The upregulated and downregulated differential genes exhibited similar characteristics in length distribution, with a significantly higher proportion of eccDNAs <1,000 bp. Analysis of the distribution of differentially expressed eccDNAs across all chromosomes indicated that the upregulated and downregulated differential genes were more abundant on chromosomes 1 and 2 and less distributed on the Y chromosome (Figure 3F). A comprehensive analysis of the Manhattan plot revealed that the upregulated differential genes exhibited a stronger correlation with HCC than the downregulated differential genes. Additionally, fewer genes were enriched on the Y-chromosomes in both groups, and their association with HCC appeared weaker (Figures 3G, H). The length distribution, chromosomal distribution, and enriched genes of differential genes aligned with the trends observed in the entire gene set.

### 3.4 Joint analysis of eccDNAs and mRNAs

**FIGURE 4:**
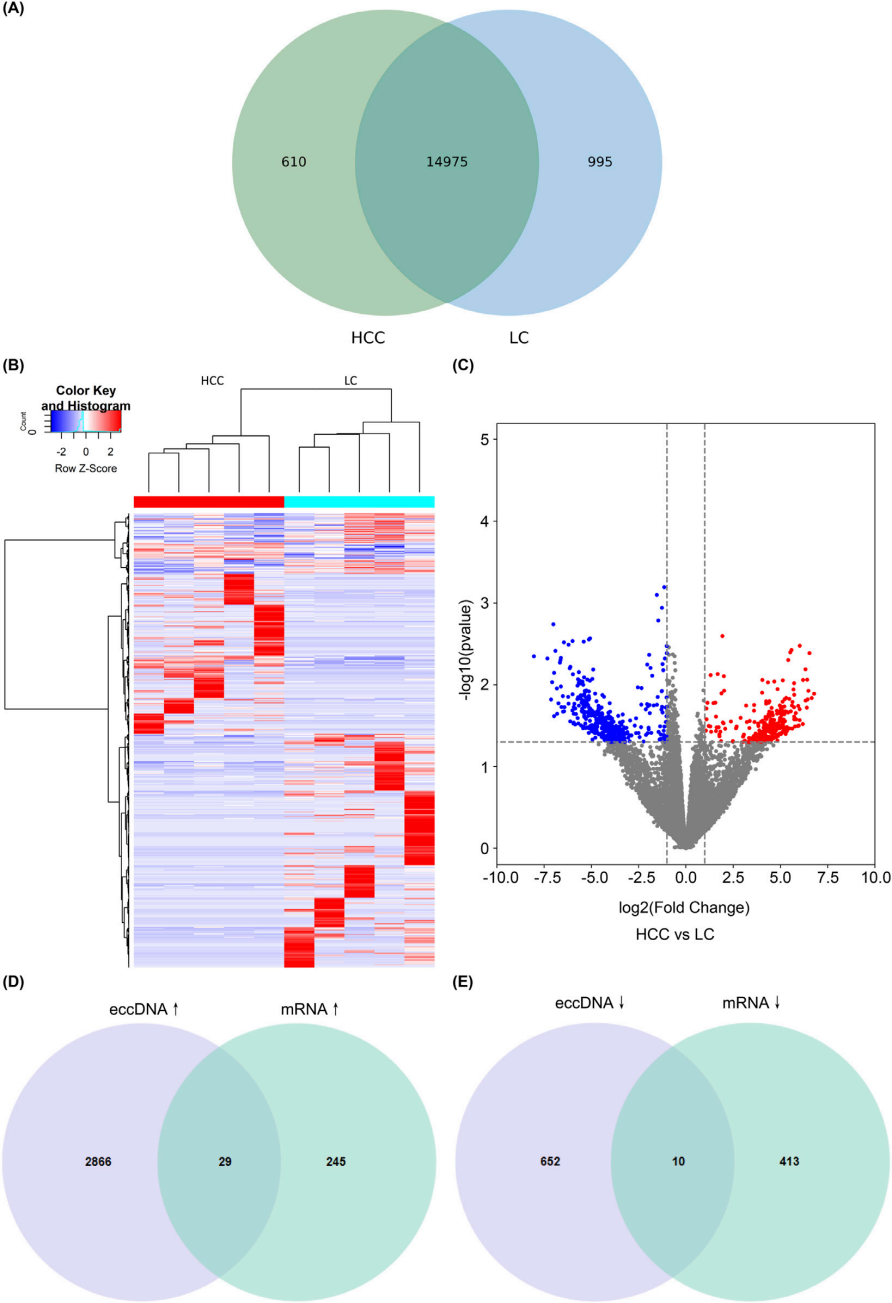
Combined analysis of eccDNAs and mRNAs. Venn diagram showing common and differential mRNAs between HCC and LC **(A)**. Cluster heatmap showing differentially expressed mRNAs in HCC and LC samples **(B)**. Volcano plots showing differentially expressed mRNAs; blue dots represent downregulated genes, red dots represent upregulated genes, and black dots represent genes with no significant difference **(C)**. Genes exhibiting overlap between eccDNAs and mRNAs are differentially expressed. The differential and overlapping genes show a co-upregulated pattern **(D)**, and differential and overlapping genes are co-downregulated **(E)**. eccDNA, extrachromosomal DNA; HCC, hepatocellular carcinoma; LC, liver cirrhosis.

We also identified differentially expressed genes between the HCC and LC groups through RNA high-throughput sequencing analysis, revealing that the two groups had 14,975 identical mRNAs and 610 and 995 mRNAs were unique to the HCC and LC groups, respectively (Figures 4A, B). The volcano plot depicts the upregulated and downregulated mRNAs (FC > 2 and *P* < 0.05; Figure 4C). Additionally, we performed a combined analysis of eccDNAs and mRNAs results, revealing that 39 candidate genes exhibited significant overlap (*P* < 0.05 in both sequencing results). Among these, 29 genes exhibited joint upregulation (Figure 4D), and 10 genes displayed joint downregulation (Figure 4E). Subsequently, we performed further functional analysis on these candidate genes.

### 3.5 Bioinformatics analysis of eccDNAs

**FIGURE 5:**
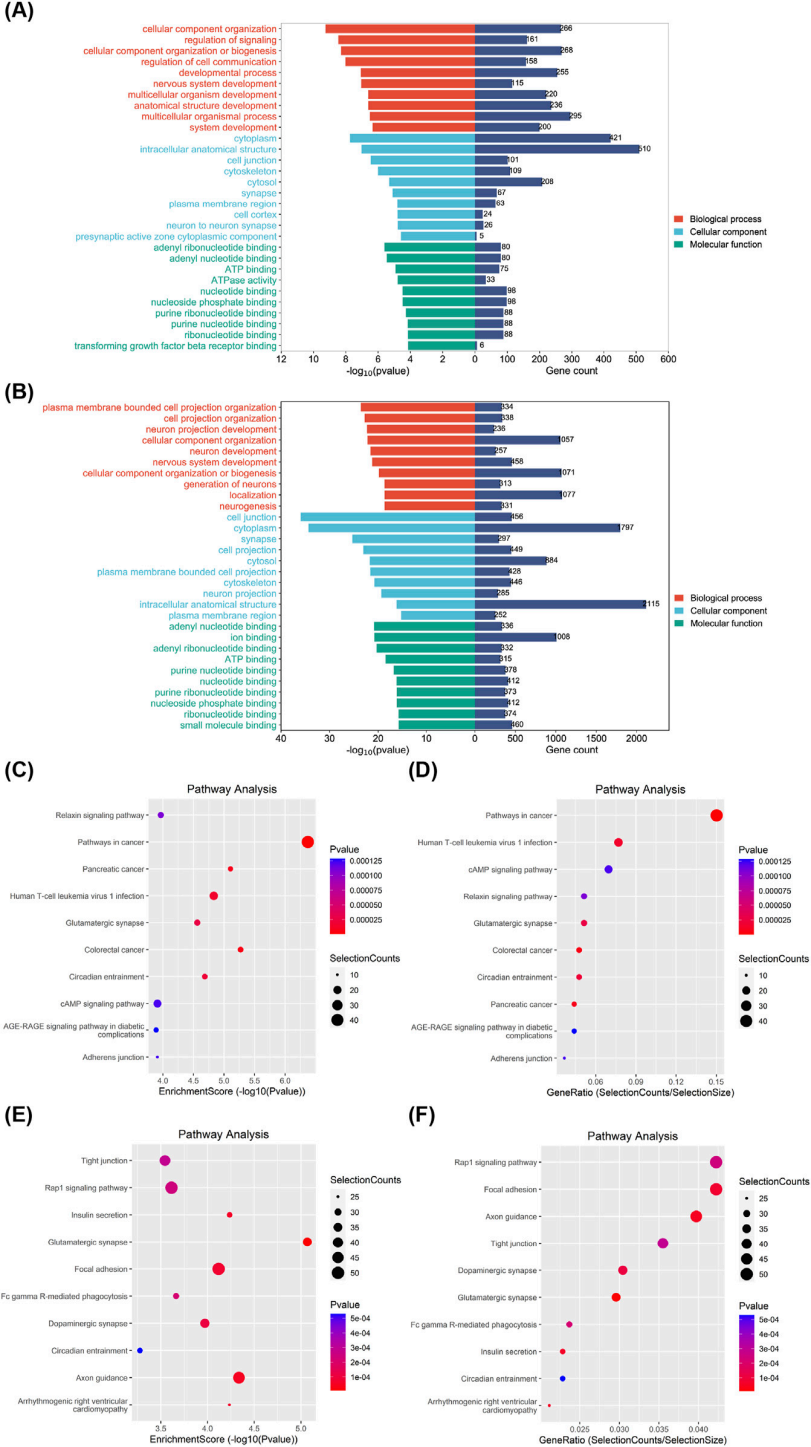
Bioinformatics analysis of differentially expressed eccDNAs in gene sets and signaling pathways. GO classification of upregulated **(A)** and downregulated differential genes **(B)** in biological processes, cellular components, and molecular functions in patients with HCC and LC and the corresponding number of genes. The top 10 most correlated pathways among upregulated **(C)** and downregulated differential genes **(E)** in KEGG enriched signaling pathways. Top 10 pathways with the largest number of upregulated **(D)** and downregulated differential genes **(F)** among the KEGG enriched signaling pathways. eccDNA, extrachromosomal DNA; HCC, hepatocellular carcinoma; KEGG, Kyoto Encyclopedia of Genes and Genomes; LC, liver cirrhosis.

We performed GO and KEGG analyses on these differentially expressed eccDNAs. Cellular component categories, molecular functions, and biological processes in GO analysis indicated that eccDNA-related genes play a key role in signal transduction, growth, and development (Figures 5A, B). KEGG pathway enrichment analysis, performed to determine the functional characteristics, revealed that both the enrichment degree and quantity of related genes were closely associated with the pathways in cancer and are closely related to the occurrence, development, and cancer-related metastasis pathways of other cancers (Figures 5C–F). The results indicated that the most significant potential downstream functions of these candidate genes are related to cancer development, metastasis pathways, and signal transduction. We selected six eccDNAs for further experiments based on the range of eccDNA and the predicted cancer-relevant functions identified through bioinformatics analysis.

### 3.6 PCR and sanger sequencing to verify the existence and expression of differential gene eccDNAs in HCC

**FIGURE 6:**
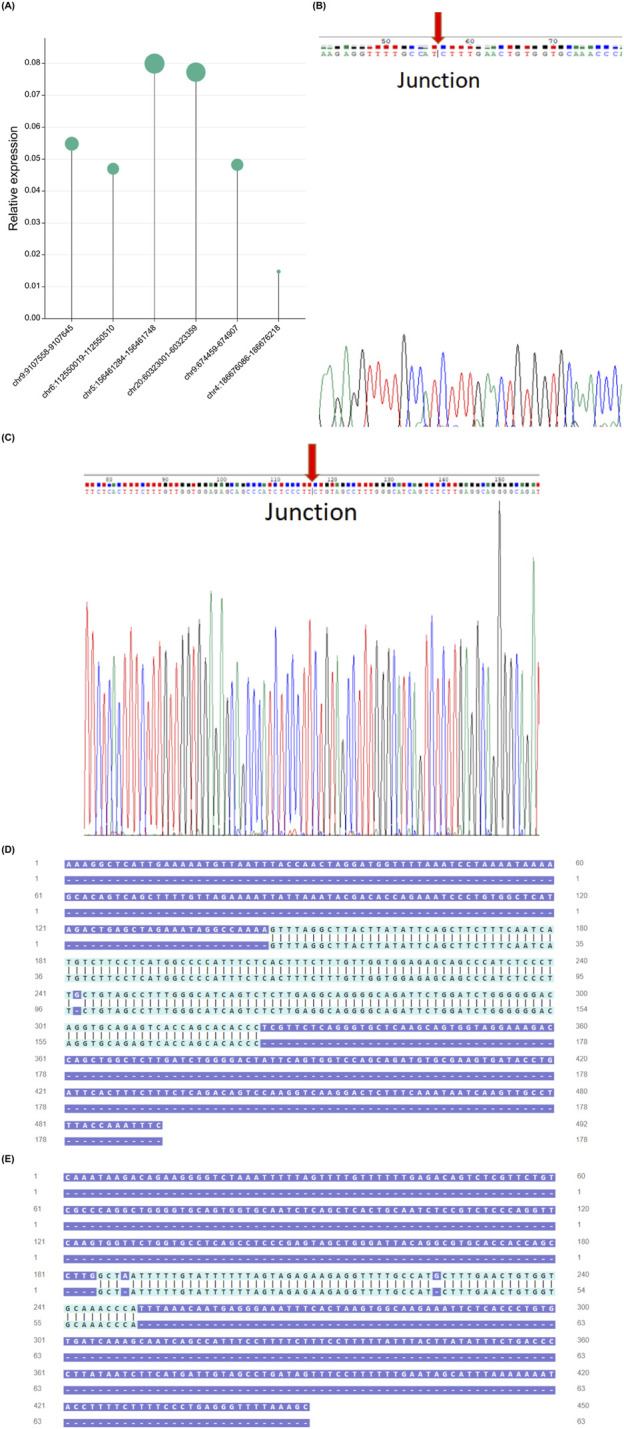
Validation of six candidate genes. Expression levels of six candidate genes in HCC samples. **(A)**. Connection sites obtained from PCR product sequencing were validated through Sanger sequencing, confirming the chr9:674,459-674907 connection site **(B)** and the chr6:112550019-112550510 connection site **(C)**. Gene chr6:112550019-112550510 **(D)**, chr9:674,459-674907. **(E)** Comparison of high-throughput sequencing PCR theoretical product and Sanger sequencing sequence. HCC, hepatocellular carcinoma; PCR, polymerase chain reaction.

Based on the predicted cancer-related functions through bioinformatics analysis and the characteristics of eccDNA, we have selected six eccDNAs for further investigation (Table 1). To validate these predicted eccDNAs, we performed reverse PCR on specific primers at six candidate gene-associated sites. All amplified products were separated and observed on an electrophoretic gel, showing accurate alignment with the expected product sizes. The expression levels of the six candidate genes significantly differed (Figure 6A). Subsequently, we used Sanger sequencing to confirm the connection site of eccDNAs. The gene at chr20:60323001-60323359 exhibited a PCR-amplified band; however, upon testing, the corresponding connection site was not detected. Similarly, genes at chr9:9107558-9107645, chr5:156461284-156461748, and chr4:186676086-186676218 produced PCR amplification products but did not correspond to the expected connection sites. This result suggests the possibility of the absence of an actual circular gene. We then conducted a sequence composition comparison between the theoretical products from high-throughput sequencing PCR and Sanger sequencing. Genes at chr9:674459-674907 (Figures 6C, E) and chr6:112550019-112550510 (Figures 6B, D) displayed consistent junction sites upon Sanger sequencing, with sequences matching the expected results. Moreover, they an overlapping sequence, which is the target circular DNA. The target eccDNAs are named *LAMA4* ^[circle112550019-112550510]^ and *KANK1* ^[circle674459-674907]^ after their source genes (Møller et al., 2018).

**TABLE 1:** Six candidate eccDNAs with differential expression of both eccDNA and mRNA.

Chr	Start	End	Size (bp)	Gene present	*P-*value	Overlap	mRNA LC vs. HCC	eccDNA LC vs. HCC
Chr9	9107558	9107645	87	PTPRD	0.049	87	↑	↑
Chr6	112550019	112550510	491	LAMA4	0.043	491	↑	↑
Chr5	156461284	156461748	464	HAVCR1	0.048	464	↑	↑
Chr20	60323001	60323359	358	CDH4	0.011	358	↑	↑
Chr9	674459	674907	448	KANK1	0.036	448	↑	↑
Chr4	186676086	186676218	132	SORBS2	0.044	132	↑	↑

ANOVA, analysis of variance; CC, cell component; EccDNA, extrachromosomal circular DNA; GO, Gene Ontology; HCC, hepatocellular carcinoma; KEGG, Kyoto Encyclopedia of Genes and Genomes; LC, liver cirrhosis; LINE, long interspersed nuclear elements; LTR, long terminal repeats; PCR, polymerase chain reaction; SINE, short interspersed nuclear elements; UTR, untranslated region.

### 3.7 Survival curve

**FIGURE 7:**
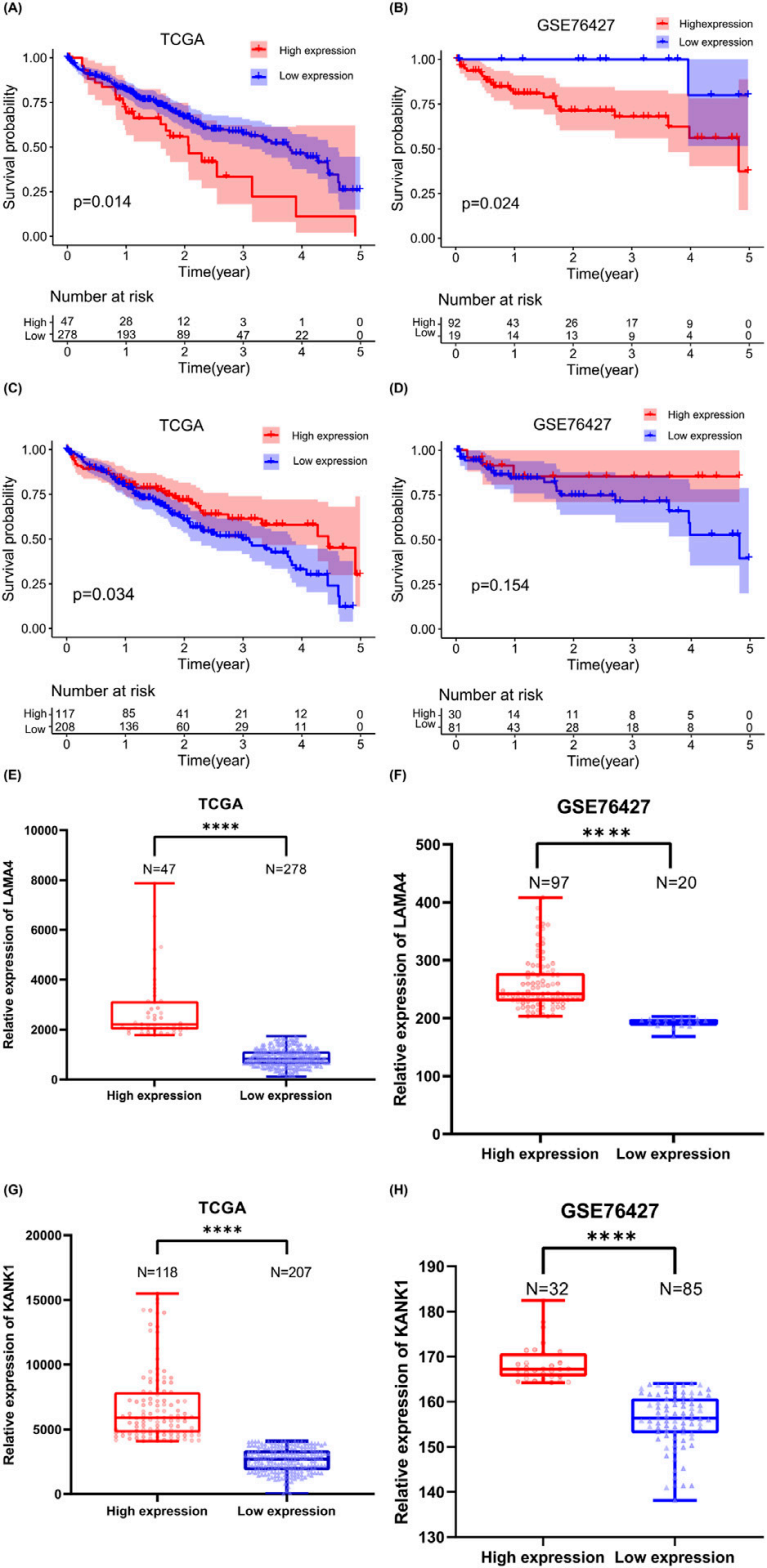
Analyzing the survival and expression levels of source genes based on TCGA and GEO databases. Kaplan–Meier analysis of the survival time **(A)** and expression level **(E)** of patients with liver cancer with high or low expression of *LAMA4* in the TCGA database. Kaplan–Meier analysis of the survival period **(B)** and expression level **(F)** of patients with liver cancer with high or low expression of *LAMA4* in the GSE76427 database. Kaplan–Meier analysis of survival time **(C)** and expression level **(G)** of patients with liver cancer with *KANK1* high or low expression in TCGA database, ****P < 0.0001. Kaplan–Meier analysis of the survival time **(D)** and expression level **(H)** of patients with liver cancer with *KANK1* high or low expression in the GSE76427 database, ****P < 0.0001.

The relationship between *LAMA4* and *KANK1* expression levels and the prognosis of patients with HCC was further analyzed using TCGA and GEO online databases. Kaplan–Meier survival analysis results of data derived from TCGA (Liu et al., 2020) and GSE7642 (Grinchuk et al., 2018) (a subset of liver cancer in the GEO database) indicated that high *LAMA4* expression (Figures 7E, F) is associated with the survival of patients with HCC with short-term correlation (Figures 7A, B). Moreover, *LAMA4* ^[circle112550019-112550510]^ and *LAMA4* mRNA expression were increased in metastatic tumors of individuals with HCC, and *LAMA4* expression level was negatively correlated with patient survival time. The higher the expression of *LAMA4* in HCC, the poorer the prognosis of patients, suggesting that *LAMA4* ^[circle112550019-112550510]^ derived from *LAMA4* may have a similar impact and could serve as a novel tumor marker for predicting future cancer occurrence.

Kaplan–Meier survival analysis using TCGA data indicated that low *KANK1* expression (Figure 7G) was associated with shorter survival in patients with HCC (Figure 7C). However, when we performed Kaplan–Meier survival analysis with GSE76427 data, no significant difference was observed between *KANK1* expression levels (Figure 7H) and survival time (Figure 7D). Both *KANK1* ^[circle674459-674907]^ and *KANK1* mRNA exhibited increased expression in metastatic HCC tumors. Thus, we *KANK1* ^[circle674459-674907]^ may also have a role in cancer prognostic assessment.

## 4 Discussion

Most patients with HBV-related liver cancer often suffer from the pathological phenomenon of cirrhosis, which makes the pathogenesis of liver cancer more complex, and patients with this type of liver cancer usually have a worse prognosis (Zhou et al., 2020). eccDNA enhances the transcription of many oncogenes by increasing DNA copy number and enabling long-distance chromatin, ultimately promoting cancer growth (Morton et al., 2019; Bailey et al., 2020). Moreover, studies have found that eccDNA is widely present in the blood system by being released into the blood circulation (Khatami et al., 2018). Because they are insensitive to digestion by exonucleases, sample acquisition is easy and easy to operate, and compared with ordinary chromosomal DNA, eccDNA has more stability. eccDNA has been predicted to become a potential biomarker in multiple diseases (Dennin and Wo, 2019; Pang et al., 2022; Zeng et al., 2022; Peng et al., 2023), indicating that it is not only functionally important, but also more suitable as a potential biomarker for early detection, risk assessment, and prognosis assessment of diseases.

In the present study, we expand our understanding of eccDNA expression in whole blood in the context of HCC and LC. We achieved the first eccDNA detection in the blood of patients with HCC and LC using Circle-seq, which enabled the establishment of the expression profiles for these conditions. We also confirmed the presence of numerous eccDNAs in both HCC and LC samples. Although HCC samples exhibited a significantly higher number of enriched variant genes than LC samples, the count of enriched eccDNAs did not strictly correlate with the number of enriched variant genes per MB, suggesting that eccDNA does not adhere to traditional chromosomal inheritance patterns (Verhaak et al., 2019). While there was no evident difference in the length distribution between the two groups, a substantial disparity was observed in the total length. Most of the lengths in both groups were <1,000 bp, aligning with the length distribution patterns observed in the blood of patients with other types of tumors (Xu G. et al., 2022). Other studies have analyzed preoperative and postoperative blood samples from patients with lung and ovarian cancer, revealing a notable rise in eccDNAs <1,000 bp in postoperative samples. This indicates that the surgical removal of cancerous tissue impacts changes in eccDNA levels (Kumar et al., 2017), and these specific eccDNAs could potentially serve as prognostic markers for patients with cancer. These data offer evidence-based support for considering small-sized eccDNAs as biomarkers for diagnosis, treatment, and prognosis.

Our findings revealed that eccDNAs can be mapped to any region of the human genome, and their formation is not dependent on the gene density of chromosomes. Importantly, chromosome 19, which is gene-rich, displays a lower frequency of eccDNA formation than chromosomes 1 and 2, where a higher number of eccDNAs are observed. The occurrence of eccDNAs mapping to the Y chromosome is notably the lowest, a characteristic shared with other cancer types (Sun et al., 2021). This disparity may be attributed to the relatively less genetic information or a denser structure of the Y chromosome. Localizing eccDNA to different genomic regions demonstrates that eccDNA is highly enriched in the 5′UTR and Alu regions but less enriched in the exon and intron regions, a pattern that diverges from previous studies (Sin et al., 2020). Simultaneously, eccDNAs are significantly concentrated within repetitive regions, such as LINE, SINE, and LTR. The proportion of eccDNAs originating from these three repetitive elements is considerably smaller in the blood of healthy individuals (Liu et al., 2020), and this variation may be influenced by chromosomal tandem repeat sequences (Cohen and Segal, 2009). It may represent a characteristic of diseases associated with HBV.

Our analysis revealed a total of 7,095 upregulated and 1,284 downregulated genes. We explored the distribution characteristics of differentially expressed eccDNAs for the first time. We found that the length distribution of differential genes and the distribution and enrichment of genes across chromosomes align closely with the overall gene trends. GO analysis revealed that the differential genes primarily function in key roles related to signal transduction, growth, and development. KEGG pathway enrichment analysis showed a strong association between these differential genes and the occurrence and development of cancer, particularly cancer-related metastasis pathways. Among these pathways, the Rap1 signaling pathway, with the most downregulated differential gene enrichment, was identified as a key factor inhibiting liver cancer development by participating in regulating metabolic transcriptional activities (Ferrara-Romeo et al., 2018). Collectively, these findings substantiate the influence of differentially expressed eccDNA on the occurrence and development of HCC.

We conducted a comprehensive analysis that integrated Circle-seq results and RNA high-throughput sequencing data, identifying certain genes with high expression levels in both eccDNA and RNA. Moreover, by employing a combination of bioinformatics analysis and Sanger sequencing, we successfully identified two novel eccDNAs, designated as *LAMA4* ^[circle112550019-112550510]^ and *KANK1* ^[circle674459-674907]^. *LAMA4* ^[circle112550019-112550510]^ is formed by the circularization of a DNA fragment within *LAMA4*. *LAMA4* plays a role in promoting tumor cell proliferation and migration, and increased *LAMA4* expression has been associated with adverse survival outcomes in HCC, pancreatic cancer, and gastric cancer (Huang et al., 2008; Wang et al., 2018; Zheng et al., 2020). Moreover, our survival analysis performed in the TCGA and GEO online databases revealed a consistent finding: high *LAMA4* expression was associated with shorter survival in patients with HCC. This observation suggests that *LAMA4* ^[circle112550019-112550510]^ may have similar diagnostic and prognostic characteristics with the *LAMA4* gene. Another eccDNA identified as *KANK1* ^[circle674459-674907]^ arises from the circularization of *KANK1* fragments. The parent gene, *KANK1*, exerts its anti-tumor effect by promoting tumor cell apoptosis (Cui et al., 2017). *KANK1* is associated with improved prognosis among patients with invasive breast cancer (Guo et al., 2014). Our survival analysis performed using the TCGA database revealed that low *KANK1* expression was associated with short survival among patients with HCC. Our findings indicate that the expression of both *KANK1* ^[circle674459-674907]^ and *KANK1* mRNA is increased in HCC, suggesting that *KANK1* ^[circle674459-674907]^ may also have a prognostic role. Although our research provides a novel direction for HCC diagnosis and treatment, the impact of *LAMA4* ^[circle112550019-112550510]^ and *KANK1* ^[circle674459-674907]^ on HCC occurrence and development still require further experimental verification.

Our study had some limitations. First, our clinical sample size is relatively small, hindering our ability to conduct correlation studies between eccDNA and clinical indicators. Second, it is essential to consider the unique sexual dimorphism of liver disease (Xu L. et al., 2022; Kasarinaite et al., 2023), where men are more susceptible to liver-related conditions than women. Hence, there exists an imbalance in the sex distribution among individuals with cirrhosis and liver cancer, with relatively few female cases. Third, during the data collection phase, the absence of female patients led to a dataset exclusively composed of male patients, potentially introducing limitations in our results. We will continue to incorporate clinical samples to enhance the eccDNA expression profile in future investigations.

In conclusion, this study revealed the expression profile of eccDNA in HBV-related liver cancer and cirrhosis, ultimately identifying the target genes *LAMA4* ^[circle112550019-112550510]^ and *KANK1* ^[circle674459-674907]^. The two target genes hold promise as biomarkers for predicting the diagnosis and prognosis of patients with HCC. Our future efforts will focus on the *in vivo* and *in vitro* experimental verification of the target genes. Additionally, we will expand sample sizes to explore the relationship between the identified genes as potential biomarkers and various clinical indicators, which would validate our hypothesis regarding the role of eccDNAs in HCC.

## Data Availability

The data presented in the study are deposited in the GEO database, accession numbers GSE271570 and GSE271574.
