# Supplementary material for: Circle-seq reveals that eccDNA may be a key blood biomarker for HBV-associated liver cancer
Source: Front Genet. 2025 Jan 9;15:1454153. doi: 10.3389/fgene.2024.1454153 (PMC11754267; doi:10.3389/fgene.2024.1454153)
Supplement: Supplementary file 1 [file Table1.DOCX]

Supplementary Material

# Supplementary Tables

**Supplementary Table 1.** PCR primers.

| **Gene name** | **Primer type** | **Gene sequence** |
| --- | --- | --- |
| chr9：9107558-9107645 | Forward | GAGAGAATCAGGCTGCCAAC |
|  | Reverse | TGTGCACACACAGACTCCTG |
| chr6：112550019-112550510 | Forward | CAGAAATCCCTGTGGCTCAT |
|  | Reverse | GAGGGTGTGCTGGTGACTCT |
| chr5：156461284-156461748 | Forward | CTCAGTGGGGAGAAACCTTG |
|  | Reverse | AGGCACAGCACCTTTCCTTA |
| chr20：60323001-60323359 | Forward | CCTCCCGGGTTTAAGTGATT |
|  | Reverse | AAGCATCCAGGTGCTGTTTT |
| chr9：674459-674907 | Forward | CCCGAGTAGCTGGGATTACA |
|  | Reverse | AATGGGTTTGCACCACAGTT |
| chr4：186676086-186676218 | Forward | CTGGTCTTCCAGGCAATCAT |
|  | Reverse | GACTCAGGGGATGTGAAGGA |
